# Fibroblast heterogeneity: Keystone of tissue homeostasis and pathology in inflammation and ageing

**DOI:** 10.3389/fimmu.2023.1137659

**Published:** 2023-02-28

**Authors:** Vincent Gauthier, Maria Kyriazi, Meriam Nefla, Valentina Pucino, Karim Raza, Christopher D. Buckley, Ghada Alsaleh

**Affiliations:** ^1^ Botnar Institute for Musculoskeletal Sciences, Nuffield Department of Orthopaedics, Rheumatology and Musculoskeletal Sciences (NDORMS), University of Oxford, Oxford, United Kingdom; ^2^ The Kennedy Institute of Rheumatology, Nuffield Department of Orthopaedics, Rheumatology and Musculoskeletal Sciences (NDORMS), University of Oxford, Oxford, United Kingdom; ^3^ Institute of Inflammation and Ageing, College of Medical and Dental Sciences, University of Birmingham, Birmingham, United Kingdom; ^4^ Faculty of Medical Sciences, Newcastle University, Newcastle-upon-Tyne, United Kingdom; ^5^ Department of Rheumatology, Sandwell and West, Birmingham Hospitals NHS Trust, Birmingham, United Kingdom

**Keywords:** fibroblast, health, diseases, ageing, inflammation

## Abstract

Fibroblasts, derived from the embryonic mesenchyme, are a diverse array of cells with roles in development, homeostasis, repair, and disease across tissues. In doing so, fibroblasts maintain micro-environmental homeostasis and create tissue niches by producing a complex extracellular matrix (ECM) including various structural proteins. Although long considered phenotypically homogenous and functionally identical, the emergence of novel technologies such as single cell transcriptomics has allowed the identification of different phenotypic and cellular states to be attributed to fibroblasts, highlighting their role in tissue regulation and inflammation. Therefore, fibroblasts are now recognised as central actors in many diseases, increasing the need to discover new therapies targeting those cells. Herein, we review the phenotypic heterogeneity and functionality of these cells and their roles in health and disease.

## 1 Introduction

Following their discovery in 1858 by Rudolf Virchow, and their description as fibroblasts by Ernst Ziegler in 1895, fibroblasts have been observed in anatomically diverse connective tissues, with a distinct spindle-shaped morphology, delineating them from the other structural cells (1–3). Their characterization has been historically driven by their distinct morphology and the absence of leucocyte, epithelial and vascular lineage markers (4). Identifying the functionality and prevalence of distinct populations of fibroblasts has led to an elucidation of their differential roles in pathological states and insights into how they may be therapeutically targeted. In the following sections, we summarize the origins, tissue-specific types, heterogeneity, and function of fibroblasts identified to date, with a primary focus on their roles in inflammation and ageing. We illustrate how impairments in fibroblast biology contribute to tissue and organismal ageing and examine how recent advances set the groundwork for therapeutically targeting these cells.

## 2 Origins and functional heterogeneity of fibroblasts across the tissues

### 2.1 Origins and tissue-specific types

**Figure 1:**
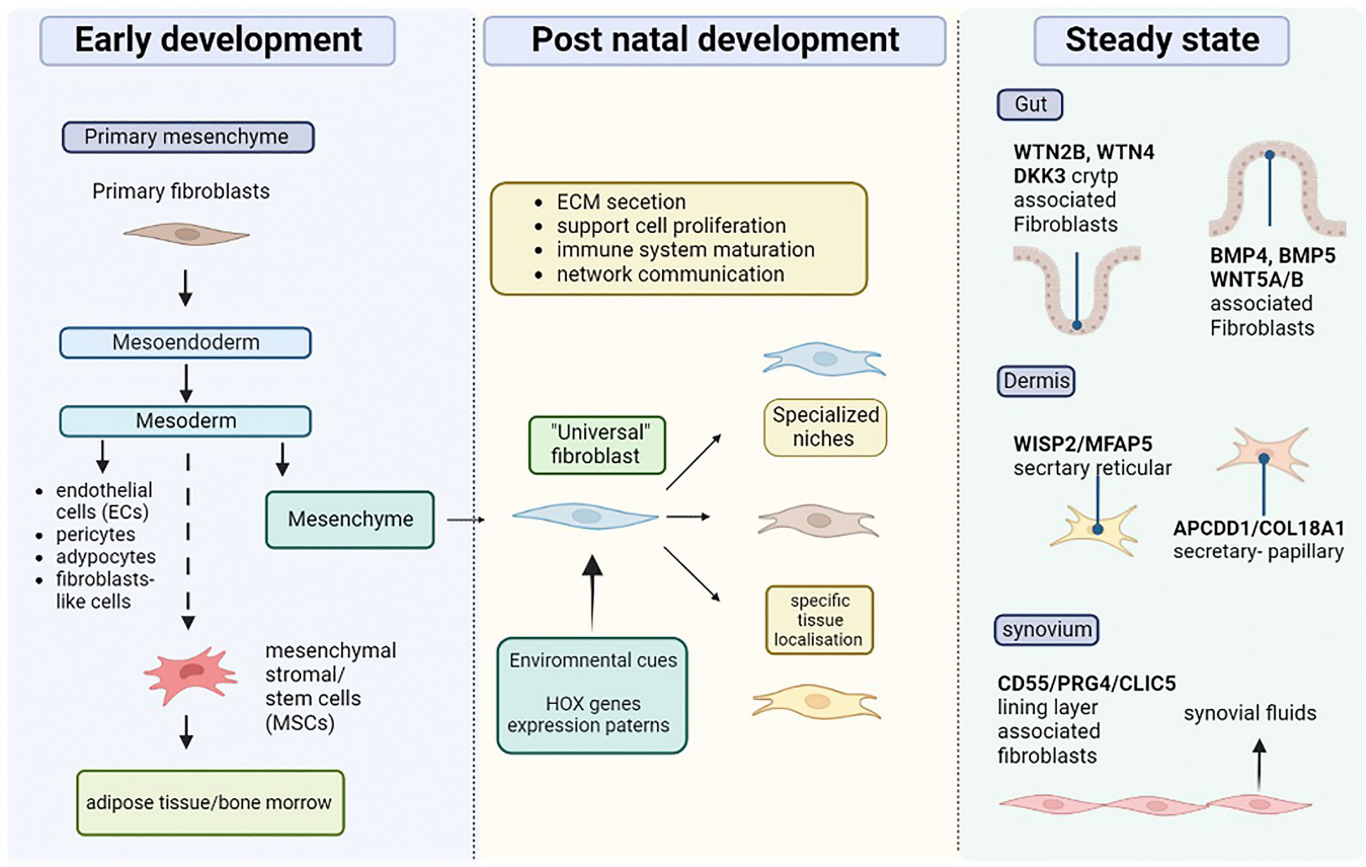
Origin and diversity of tissue resident fibroblast. During the early stages of development, the primary mesenchyme is mad up of primary fibro plats, which eventually give rise to the mesoendoderm. The mesoderm also gives rise to mesenchyme stromal or stem cells (MSCs), which can populate specific areas such as the bone marrow and adipose tissue. In addition, the mesoderm produces endothelial cells (ECs), adipocytes, and mesenchyme, which is where resident fibroblasts originate. Environmental cues and transcription of HOX genes give tissue localization instruction to resident fibroblasts which leads to their specific roles. Fibroblasts populate specific niches in different tissue supporting roles including cell proliferation, ECM secretion, immune cells, and maturation. According to the tissue and environmental needs fibroblasts develop specific function, (e.g., synovial fluid secretion).

Recent technological advances have allowed a better understanding of the origin of fibroblast (summarised in **Figure 1**). During early embryonic development, gastrulation forms three primary layers, so-called ectoderm, mesoderm, and endoderm. These layers give rise to specific tissues and organs in the developing embryo. During the process of gastrulation, the epiblast gives rise to two main cell types: the primary mesenchyme and the ectoderm. The primary mesenchyme is made up of primary fibroblasts, and further differentiation of these cells leads to the development of the endoderm and mesoderm. The mesoderm gives rise to various cell types, including endothelial cells, pericytes, adipocytes, and mesenchyme. Mesenchymal stromal cells may also develop from the mesoderm and help create a supportive environment in certain tissues. In adults, the mesenchyme becomes quiescent fibroblasts known as resident quiescent fibroblasts (RQF). Together, epithelial cells, endothelial cells, perivascular cells, adipocytes, and fibrocytes from the bone marrow, participate in the generation of fibroblast-like cells during injury through processes known as Type 2 and Type 3 epithelial-to-mesenchymal transitions (EMT) (5). In adults the mesenchyme comprises resident fibroblasts that support ECM formation and remain quiescent in the absence of stimulation. Thus, fibroblasts are responsible for tissue homeostasis and contribute to extrinsic tissue ageing (5). Transcriptional regulation determines the localization of resident fibroblasts and defines their distribution through development. Additionally, fibroblasts are imprinted with positional identities laid down during development and maintained by the epigenetic regulation of homeobox transcription factors, so-called HOX genes, responsible for positional patterning during development (6, 7). Although all fibroblast subsets share a mesenchymal origin, their differences rely upon their localization, functionality, and cellular state. The different fibroblast types defined by their tissue specific gene enrichment and marker expression allows the depiction of fibroblast subtypes in tissues including the heart, gut, lungs, colon, skin, and joints. Cardiac/Myocardial fibroblasts express atrial and ventricular markers (8). Fibroblasts found in the lungs comprise lipofibroblasts (LipFB), myofibroblasts (MyoFB), alveolar (AlvFB) and adventitial fibroblasts (AdvFB) (9). The skin includes three fibroblast subtypes, type A (FB-A), responsible for dermal cell and ECM homeostasis, type B (FB-B), regulating immune surveillance and inflammation and type C (FB-C), comprising specialized subpopulations (10). Recent evidence also suggests the expression of myofibroblasts and pericytes, as well as a distinct population of fibroblast-like cells expressed in the colon that are associated with health status or with the development of inflammatory bowel disease (IBD) and colitis (11). Gut fibroblasts, including interstitial fibroblasts, are characterized by the expression of differential markers depending on their localization within the gut (e.g., villus, crypt). In healthy adult joints, fibroblast-like synoviocytes here termed synovial fibroblasts (SFs) are observed in both the synovial lining layer (LL) and the sub-lining layer (SL), expressing location-specific proteins and cytokines, and associated with either inflammatory or destructive diseases. Therefore, one could logically think that different fibroblast subsets exist in both healthy and diseased states. Indeed, recent data published by Buechler et al. indicate that universal and specialized fibroblast subsets, so-called steady state subsets, can exist together with activated, perturbed state subsets, within the same tissue (12). Understanding the different fibroblast markers observed in steady and perturbed states is thus likely to further our understanding of the mechanistic roles of these cells in the co-existing microenvironment.

### 2.2 Heterogeneity across the tissues

Fibroblasts are characterized by their secretion of ECM molecules including elastin, fibronectin, periostin, laminins, proteoglycans, microfibrillar proteins, and fiber- or sheet-forming collagens, creating the “matrisome” and which in turn influences their function. Since no totally lineage specific markers have been identified, fibroblasts are captured using archetypal mesenchymal markers, including vimentin, PDGFRα/β (platelet-derived growth factor receptor-alpha and -beta), PDPN (podoplanin), CD90 (THY-1), FAP (fibroblast activation protein) marking activated fibroblasts, and αSMA (α-smooth muscle actin) typically expressed in myofibroblasts, the fibroblast type that is activated in response to injury and is responsible for the tissue repair (5, 13–15). However, these markers are not ubiquitously expressed upon all the fibroblasts, diminishing our understanding of the heterogeneity of this cell type.

Recent technological advances, including single cell RNA sequencing (scRNA-seq) have begun to reveal fibroblast heterogeneity and provide insights into their roles in disease (**Table 1**). However, whether subtype heterogeneity emerges from broad transcriptional programming (intrinsic triggers), or from other cell types in an extrinsic context dependant manner still remains to be established. A recent study in a murine model suggests the existence of *dpt+* universal fibroblasts providing functional plasticity to activated fibroblasts across tissues (12). This supports the idea that fibroblast phenotype depends on their activation state in addition to potential tissue-specific cues. The authors propose that fibroblasts exist in a universal and specialized state, called steady state, or an activated state during perturbation (inflammation, wound healing, fibrosis, or cancer). Consequently, fibroblast identity changes depending on the surrounding environment.

**Table 1:** Fibroblasts subsets identified in health or disease and their localization in the tissues.

Fibroblasts subsets	Tissues	Localization	Healthy	Diseases	Function	References
THY1+/HLA-DR^HI^	Synovium	Sublining/perivascular	No	RA	Inflammation in RA	Zhang et al. (16)
						Croft et al. (17)
CD55+PRG4+CLIC5+	Synovium	Lining layer	Yes	Disturbed in RA	Synovial fluid secretionBone and cartilage damage in RA	Zhang et al. (16)Croft et al. (57°
ACTA2+ myofibroblasts	lung	Sub-epithelial regions of large airways	Yes	Expended in pulmonary fibrosis	ECM secretion	Habermann et al. (18)
PLIN2+	lung	Interstitial region	Yes	Expended in pulmonary fibrosis	Phospholipid storage	Habermann et al. (18)
WISP2+MFAP5+	Skin	Reticular dermis (RD)	Yes	Not defined	structural and ECM organization	Janson et al. (19)
APCDD1+ COL18A1+	Skin	Papillary dermis (PD)	Yes	Not defined	Structural and ECM organization	Janson et al. (19)
COL6A5+ COL18A1+	Skin	Lesion sites	No	Atopic dermatitis	Inflammation	He et al. (20)
WTN2B+ WTN4+ RSPO3^+^	Gut	Crypt	Yes	Not defined	Support intestinal stem cell niche via LCR5	Smillie et al. (21)
BMP4+ BMP5+ WNT5A/B+	Gut	Villi	Yes	Not defined	Epithelium formation	Smillie et al. (21)
APCDD1+ COL18A1+	Skin	Papillary dermis (PD)	Yes	Not defined	Structural and ECM organization	Janson et al. (19)
COL6A5+ COL18A1+	Skin	Lesion sites	No	Atopic dermatitis	Inflammation	He et al. (20)
WTN2B+ WTN4+ RSPO3^+^	Gut	Crypt	Yes	Not defined	Support intestinal stem cell niche via LCR5	Smillie et al. (21)
BMP4+ BMP5+ WNT5A/B+	Gut	Villi	Yes	Not defined	Epithelium formation	Smillie et al. (21)
SPARCL1+PTGDS+	Oral mucosa	Buccal mucosa	Yes	Present in periodontitis	Active translation	Williams et al. (22)
COL1A1+COL1A2+POSTN+MMP2+	Oral mucosa	Buccal and gingival mucosa	Yes	Potentially altered in periodontitis	Structural and ECM secretion/	Williams et al. (22)
					organization	
ANXA1+ IGFBP2+	Oral mucosa	Buccal and gingival mucosa	Yes	Present in periodontitis	Regulation of leukocyte proliferation	Williams et al. (22)
CXCL1, 2, 8	Oral mucosa	Buccal and gingival mucosa	Yes	Periodontitis	Granulocytes migration	Williams et al. (22)
C3+ CFD+	Oral mucosa	Buccal and gingival mucosa	Yes	Periodontitis	Inflammation and complement activation	Williams et al. (22)
PDPN+CD34+ICAM-1+VCAM-1	Salivary glands	TLS	No	Sjogren’s syndrome	Leucocyte survival	Nayar et al. (23)
PDPN+CD34-CCL19+	Salivary glands	TLS	No	Sjogren’s syndrome	TLS organization	Nayar et al. (23)

#### 2.2.1 Gut

Differential expression of the WNT/BMP pathway is observed in gut fibroblasts, providing them with location-specific properties. Indeed, WTN2B, WTN4 and DKK3 enriched fibroblasts are associated with the crypt, while BMP4, BMP5 and WNT5A/B enriched fibroblasts reside in the villus (21). These transcriptional and positional differences underline the role of fibroblasts in supporting other cell types in specific niches. In this context, a POSTN fibroblast population has been identified to support epithelium by expressing factors related to epithelial cell proliferation and maintenance (11). Similarly, within the WNT2B population, a RSPON3+ (R-spondin-3) subset supports the intestinal stem cell niche through its interaction with the LCR5 receptor, highlighting the role of fibroblasts heterogeneity in maintaining sub-compartmental function within the tissue.

#### 2.2.2 Skin and dermis

The dermis is an important regulatory layer of the skin underlining the epidermis. It principally consists of ECM secreted by numerous fibroblasts and is divided in two regions: the papillary dermis (PD) and the reticular dermis (RD) (15). As in the gut, different fibroblast populations are localized in the distinct structures of the dermis. PD fibroblasts exhibit morphological differences compared to RD fibroblasts. While PD fibroblasts are thin and spindle-shaped, RD fibroblasts have a squarer shape (19). ScRNA-seq studies on skin biopsies identified 4 clusters of dermal fibroblasts and two subpopulations with important structural and ECM organization roles. The first, in the RD (so-called secretary-reticular), is enriched with WISP2 or MFAP5. The second sub-population (so-called secretary-papillary) is found in the PD region and expresses APCDD1 or COL18A1, previously described as papillary markers. The third population expressing Asporin (ASPN) and Periostin (POSTN) is implicated in mesenchymal regulation. The final sub-population represents a pro-inflammatory state, enriched with CCL19, APOE, or CXCL2 expression (24). However, poor overlap of adult dermal fibroblasts subpopulations has been observed in other scRNA-seq datasets (20, 25–27). A comparative study on all published datasets on human skin, identified 3 mains dermal fibroblast types, type A fibroblasts implicated in ECM homeostasis and potentially in fibrotic linage, type B fibroblasts involved in inflammation and immune surveillance and type C corresponding to a specialized skin specific subpopulation such as papillary dermis fibroblasts. While disparities between datasets can be explained by variation in experimental processes, once clustered together those datasets reveal biological similarities between them. Thus, these discoveries are improving our knowledge on the heterogeneity in skin fibroblasts populations paving the way to understand potential dysregulation in diseases.

#### 2.2.3 Oral mucosa

The oral mucosa is the first site of encountered for food, airborne antigens, and commensal microbiome. The oral cavity mucosal tissue comprises multilayer squamous epithelium divided in three main regions; the masticatory mucosa (gingiva, hard palate and dorsum of the tongue), the specialized mucosa (taste buds associated), and the lining mucosa (inside the cheeks, floor of the mouth) (28, 29). Despite the constant exposure to antigens, commensals and microdamage, the oral mucosa display efficient wound healing and minimal scar formation without aberrant inflammation. This suggests meticulous regulation between the stromal compartment and the immune cells at those sites. Recently, the transcriptomic profile of gingival/oral fibroblasts reveals 5 sub populations in adult healthy gingiva and lining mucosa (22). One of the fibroblast cluster display collagen synthesis and ECM remodelling functions (COL1A1, COL1A2, MMP2). A second cluster expressing SPARCL1 display an active translation status. Finally, 3 clusters are associated with inflammatory signature genes: ANXA1+IGBP2+ enriched fibroblasts associated with leucocytes proliferation, CXCL1+CXCL2+CXCL8+ fibroblasts involved in granulocyte recruitment, and C3+CDF+ fibroblasts for the complement activation (22).

#### 2.2.4 Heart

The heart is another fibroblast-enriched organ. Cardiac fibroblasts are responsible for the ECM remodelling, which is critical for electrical conductivity and heartbeat rhythm (30). Adult cardiac fibroblasts comprise myocardial, pericardial and epicardium layers, containing specialized adipose tissue and endothelial cells, respectively (30). These populations, revealed by single-cell transcriptomic studies in mouse, comprise two main groups and their lineage contributions are distinct. For example, *Forte et al.* discovered a small endocardial-derived fibroblast population expressing Wif1 and Dkk3 WNT signalling factors, relating to valve leaflets and endochondral specification toward the bone lineage, and a larger epicardial-derived fibroblast population presenting the expression of genes associated with metabolism, and cell migration (31).While other ScRNAseq studies on murine cardiac fibroblasts suggests two major sub populations based on the differential expression of Sca1. The Sca1high and Sca1low subpopulation both expressed canonical fibroblasts markers such as PDGFRα or Col1 and seems to have distinct adhesive and secretory phenotype. Moreover, the authors also distinguish a fibroblast population highly regulated by WNT signalling (32).

#### 2.2.5 Lungs

Lung fibroblasts, like many other fibroblasts are marked by PDGFRα expression. Additional heterogeneity is observed within PDGFRα+ lung fibroblasts including a WNT subset which is also characterized by Axin2 expression (18). PDGFRα expression is increased upon Gli1+ (part of the Hedgehog signaling pathway) maturation of the Gli1+ progenitors, which label the mesenchymal lung cells, but not lipofibroblasts, and depend on Hedgehog signaling (16, 18, 33). In healthy lung fibroblasts, AXIN2+ PDGFRα+ are located towards the alveolar niche supporting the maintenance of the stem cell niche, whereas AXIN2+ PDGFRα- fibroblasts, a population associated with pathogenic remodeling in disease, are positioned towards the airways (3). In addition, αSMA+ fibroblasts express Tbx4 in response to lung injury (34).

The sub-epithelial regions of large lung airways are characterized by the expression of ACAT2+ in myofibroblasts secreting ECM and which show a dysregulated expansion in pulmonary fibrosis. The interstitial regions of the lungs express PLIN2+, responsible for phospholipid storage, a subset which are also elevated in pulmonary fibrosis (35).

#### 2.2.6 Joints

The synovial membrane is histologically separate in two compartments: the lining layer and the sub-lining. Highly specific PRG4+CD55+CLIC5+ fibroblasts, are found in the synovial lining layer and are responsible for the secretion of synovial fluid (3, 36). By contrast, in resting condition the sub-lining fibroblasts are not well defined. However, significant increase in fibroblast heterogeneity is observed in the joints of patients with active synovial inflammation, contribution to both Inflammation and bone/cartilage erosion.

In resting conditions, fibroblasts modulate specific tissue niches by adapting to the needs of the surrounding microenvironment. Increasing scRNA-seq data on human fibroblasts in healthy individuals are unmasking new functions of fibroblasts beside the archetypal ECM formation.

### 2.3 Functional characterization of fibroblasts

Fibroblasts are critical for organizing functional tissue networks, due to ECM secretion, defining the tissue architecture and allowing cells to migrate and communicate. Thus, they have been implicated in the formation of specialized niches which support various processes including stem cell proliferation, haematopoiesis, and even joint lubrication (11, 37–40). Their multifaced properties also allow them to establish the functioning and positioning of other cell types. Following tissue damage fibroblasts are responsible for tissue healing, inflammation, and scarring (40).

Among those specialized functions, fibroblasts play a dominant role in immune system establishment from addressing alert signals during inflammation and participating in the T and B cell maturation and trafficking (38, 41).

Immune cell recruitment to peripheral tissues requires specific cues from endothelial cells. This includes cell interactions through adhesion molecules, including selectins and integrins, and activation molecules called chemokines (42). Due to their immunological properties, fibroblasts play a critical role in supporting the recruitment and retention of leukocytes within the tissue (43, 44), a process, that is essential for establishing an immune response (41). Stromal niches coordinate lymphocyte trafficking and survival in lymphatic tissues (45). In lymphoid tissue, fibroblastic reticular cells (FRCs) shape the stromal architecture of the secondary lymphoid organs (SLO) secreting chemokines, survival cues and the ECM network necessary for establishing the adaptative immunity (46). FRCs originate from the mesenchymal lymphoid tissue organizer cells (mLTo), expressing PDGFRα/β, which then differentiates into specialized FRC subsets in a “2 signals” model (47). The first signal, comprising lymphotoxin-β receptor (LTβR) and nuclear factor nuclear factor-kappa B (NF-κ B), commits to the FRC lineage differentiation, whereas the second signal provides FRC-specialized identities. Those FRC-specialized subsets form distinct niches of the SLO and are characterized by their localization and immune interactors. For instance, in the lymph node, FRC landscape comprise marginal reticular cells (MRC), follicular dendritic cells (FDCs) in the dark zone and in the light zone, T‐B border reticular cells (TBRC), interfollicular reticular cells (IRC), medullary reticular cells (MedRC), T cell reticular cells (TRC), and perivascular reticular cells (PRC) around blood vessels (47). While the second signal is not yet fully understood, some molecules such as tumor necrosis factor (TNF) or receptor activator of nuclear factor kappa-B ligand (RANKL) are involved in the establishment of FDC or MRC respectively (47, 48). FRCs also support immune cell communication by establishing chemokine gradients (CXCL13, CXCL12, CXCL21, CXCL19) and survival cues including IL-7, or RANKL (46). Thus, the stromal compartment comprising fibroblasts, is essential for many immune processes by elaborating a precise set of cytokines and chemokines to drive immune cell communication.

## 3.0 Fibroblast dysregulation in disease

### 3.1 Role in the immune response: The loss of homeostatic balance leading to inflammation

As already mentioned, fibroblasts support the recruitment and activation of immune cells, by secreting and responding to cytokines, chemokines, and other inflammatory stimuli. A multi-omics, cross-tissue study has demonstrated that fibroblasts, along with endothelial and epithelial cells, rapidly respond to immune activation based on epigenetic changes (49). Inflammation relies on an orchestrated series of events comprising the recruitment of immune cells, their activation, and a final resolution phase. It now appears that fibroblasts are critical in all these phases. Therefore, dysregulation of the immunomodulatory properties of fibroblasts can lead to persistent chronic inflammation and failed resolution. The resolution of the inflammation is vital for tissue homeostasis. Eliminating pro-inflammatory and survival signals initiates inflammation resolution, which is followed by an increase in apoptotic signalling and re-entry of the remaining inflammatory effectors in the circulatory and lymphatic system (50). This process can be perturbed by fibroblasts that remain primed to inflammatory signals leading to abnormal retention of lymphocytes within the peripheral tissue. This phenomenon is observed in rheumatoid arthritis (RA), where synovial fibroblasts (SFs) are primed by inflammatory signals, increasing their inflammatory potential. Indeed, upon tumor necrosis factor alpha (TNF-α) stimulation, SFs exhibit prolonged activation of NF-κ B leading to excessive interleukin 6 (IL-6) levels (51). This effect is increased upon re-stimulation confirming the priming potential of SFs in inflammation (52). Furthermore, a recent study has demonstrated that intracellular activation of the complement C3 component in rodent SFs induces glycolysis and activates the inflammasome (53). Therefore, primed SFs produce pro-inflammatory RANKL, IL-6, TNF and IL-1β, mediating inflammatory bone destruction. Evidence has also demonstrated the role of stromal cells in activating CXCR4, a chemokine receptor supporting the infiltration of synovial T cells, subsequently increasing their retention in the tissue *via* the endothelial expression of SDF-1 (CXCR4 ligand) (54, 55).

Other chemokines are also involved in CD4+ and CD8+ T cell accumulation and migration to the synovial microenvironment, including CCL5 or CXCL11 (56). Besides chemoattractant signals, apoptotic resistance is also increased in RA through T cell interaction with SFs *via* integrins (57). Excessive lymphocyte accumulation in RA is also mediated by the expression of pro-survival SF molecules, impairing accurate resolution. For instance, synovial fibroblasts secrete interleukin 7 (IL-7) and IL-15 inducing T cell proliferation (58). Altogether, this evidence suggests that synovial fibroblasts adopt FRC properties during chronic inflammation and permit the formation of ectopic-lymphoid structures, known as tertiary lymphoid structures (TLS) (17, 23, 59). By communicating with immune cells and shaping the microenvironment toward inflammation or resolution, fibroblasts appear as active contributors to inflammatory diseases, fibrosis, or cancer. Therefore, understanding the factors that drive fibroblast heterogeneity might help therapeutic targeting of inflammatory-mediated diseases.

### 3.2 Heterogeneity in the progression of disease across tissues

#### 3.2.1 Rheumatoid arthritis

Fibroblasts cell states are dramatically altered in diseased tissues. For instance, fibroblasts in the joints of RA patients consist of discrete fibroblast populations which become pathogenic upon repetitive inflammatory signals. The synovium is a thin connective tissue at the interphase of the bones and is essential to maintain a low friction environment by secreting the synovial fluid into the synovial cavity. It comprises two distinct mesenchymal compartments: an epithelial-like lining layer formed by tissue resident macrophages and lubricin (PRG4) expressing lining layer fibroblasts and a sub-lining structure which contains fibroblasts the vascular system and the immune cells (39). During inflammation, the architecture of the sub-lining drastically changes, and the SL fibroblast population expands. Using data from the Accelerating Medicines Partnership Rheumatoid Arthritis/Systemic Lupus Erythematosus (AMP RA/SLE) Consortium, *Zhang et al.* identified 4 synovial fibroblast subsets: a perivascular and interstitial THY1+/HLA-DR^hi^, perivascular sub-lining CD34+ fibroblasts, a DKK3+/CADM1+ sub-lining population and CD55+PRG4+ lining layer fibroblasts (36). The CD55+ lining layer fibroblasts are the most transcriptionally distinct subset compared to the THY1+ sub-lining fibroblasts. Those results have been further confirming by *Croft et al.* and validated in the mouse synovium (60). *Croft et al.* also demonstrated functional heterogeneity of the fibroblast populations during arthritis. Indeed, intra-articular injection of FAP+THY+ fibroblasts resulted in severe and sustained inflammation, whereas injecting FAP+THY- increased osteoclast activity and bone damage *via* the activation of nuclear factor-κB ligand (RANKL) (60).

In RA THY1+ fibroblasts expand from the perivascular niche in the direction of the lining layer and endothelial cells provide positional cues for fibroblasts *via* NOTCH3 signalling (61). NOTCH3 activation in fibroblasts also modulates the pathogenic sub-lining phenotype and increases their expansion. Genetic depletion and antibody blockade of NOTCH3 decreases synovial inflammation and damage, suggesting the important role of endothelial-fibroblast crosstalk in RA. By contrast, the lining layer fibroblast identity is maintained in the absence of endothelial positional cues (62). However, a recent study have demonstrated a novel transcriptional regulation which direct the fate of SFs toward a ECM degrading phenotype. Indeed, *Yan et al.* identified the transcription factor ETS1 as a positive regulator of *Tnfsf11* (coding for RANKL), MMP13 and MMP3 in arthritic SFs (63). This discovery gives new lines of thought in fate decision process of pathological fibroblasts besides the identified environmental cues.

#### 3.2.2 Ulcerative colitis

In the gut, most fibroblast subsets exist in both healthy individuals and in patients with ulcerative colitis (UC), with inflammatory fibroblast populations in UC expanding (21). These inflammatory associated fibroblasts are characterised by the expression of the colitis and fibrosis related gene, IL-11, and share markers with cancers-associated fibroblasts (CAFs), such as the fibroblast activated protein (FAP), or WNT2 (63), suggesting a putative shared state/origin between them. Furthermore, OSMR (Oncostatin M) is highly expressed in the inflammatory-associated fibroblasts, highlighting their role in anti-TNF therapy resistance (64).

#### 3.2.3 Periodontitis

Periodontitis is an inflammatory disease affecting the gingiva due to a dysbiosis on the tooth surface. Transcriptional analysis on periodontitis tissue indicates a decrease of the stromal cells (endothelial/fibroblast) proportion, albeit presenting transcriptional similarities compared to healthy tissue (22). Nevertheless, a transcriptional shift toward inflammation is observed in the epithelial and stromal population including the fibroblasts clusters previously described in health. Indeed, genes associated with antimicrobial response are enriched in the fibroblast populations. Moreover, the increased expression of CXCL1, CXCL2 and CXCL3 by the fibroblasts reflect an active recruitment of neutrophil. A parallel study also demonstrated the increased of inflammatory fibroblasts proportions in mild and severe periodontitis gingiva (65). In addition, the authors report a disruption of fibroblast population known to be involved in tissue repair. Notably characterized by a diminution of collagen VI in severe periodontitis tissue (65)

#### 3.2.4 Pulmonary fibrosis

*Habermann et al.* have identified different fibroblast states in the lungs of pulmonary fibrosis patients compared to non-fibrotic donors, including ACTA2+ myofibroblasts, PLIN2+ lipofibroblast-like, HAS1 high populations (35). Overall, fibroblast populations are expanded in fibrotic lung tissue compared to controls and are located in specific niches. Indeed, the aSMA encoding *Acta2*-expressing myofibroblasts are expanded in the sub-epithelial regions of the large airways, while PLIN2+ populations are diffusely distributed in the interstitial regions around the alveoli and HAS1+ populations in the sub pleural regions. Dysregulated gene expression in these fibroblast niches promote pathologic ECM expansion.

The presence of various fibroblast populations in disease supports the concept that fibroblasts are key players in many pathologies by sustaining inflammation or increasing fibrosis. Recognising that fibroblast heterogeneity depends on their environmental cues, it is critical to determine if the activation of pathogenic fibroblast subsets is tissue-dependent or shared across diseases.

### 3.3 Cross tissue pathological implications

**Figure 2:**
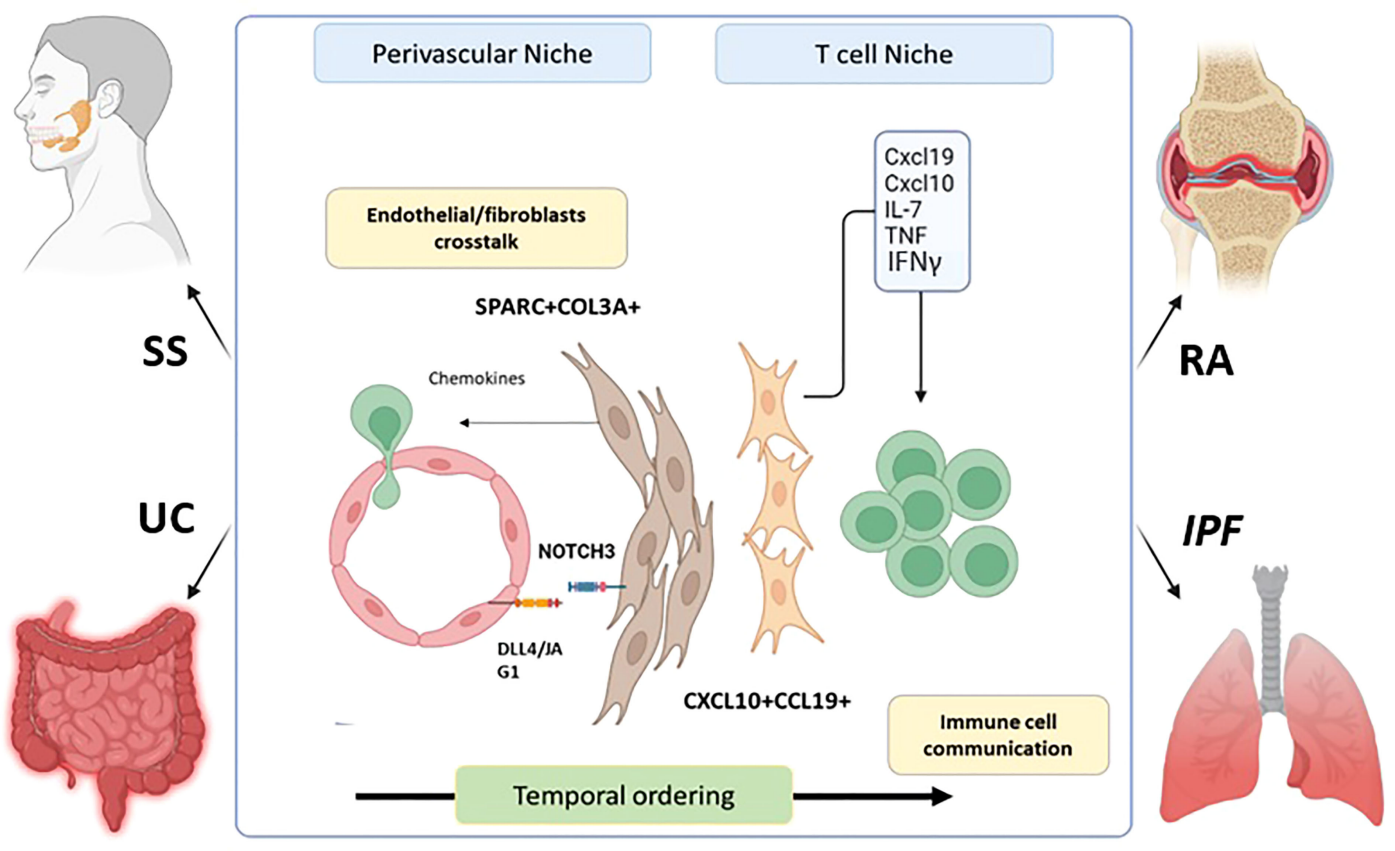
Fibroblasts shared an inflammatory phenotype across diseases and tissues. SPARC + COL3A + fibroblasts expend in the perivascular niche during inflammation after crosstalk with endothelial cells via NOTCH signaling pathway. This leads to their accumulation and the expression of chemokine’s promoting T and B cell infiltration in the tissue. Later, the CXCL10+CCL19 + fibroblasts population emerged and promote the retention and survival of T cell with in the tissue by the expression if IFNy, IL-7, TNF, and various chemokine’s. This establishment is temporal as evidence in mouse colitis models shows the transitory apparition of SPARC+COL3A+ followed by CXCL10+CCL19 + population. This mechanism was characterized in RA. However, SPARC + COL3A _ CXCL10+CCL19 + and CXCL10 +CCL19 _ fibroblasts are expended during RA (Rheumatoid Arthritis), SS (Sjogren’s syndrome), IPF(idiopathic pulmonary Fibrosis) and UC (Ulcerative Colitis).

Novel statistical models for integrative clustering of scRNA- seq datasets allow an assessment of the cellular state across species, tissues, and diseases. In line with the evidence of a universal dpt+ fibroblasts in the mouse (12), *Korsunsky et al.* elucidated the presence of shared fibroblasts activation states across human diseases and tissues (66). The authors compared the transcriptomic profiles of fibroblasts from multiple organs using individual adult scRNA-seq datasets including the Adult Human Cell Atlas (AHCA) (67) and Tabula Sapiens (TS) (68). They found over 256 genes from AHCA and 357 from TS as universal fibroblast markers across tissues, generating *de novo* scRNA-seq data to characterize inflammatory fibroblasts from the gut, the lungs, the synovium, and the salivary glands. It is important to note that this study is pioneering in its use of scRNA-seq on salivary glands from patients with Sjögren’s syndrome and confirms the presence of PDPN+CD34+ and CD34-CCL19+ populations as identified through multi-channel flow cytometry (23). Between those 4 organs the authors identified 5 clusters, with shared markers, including SPARC+COL3A1+, FBLN1+, PTGS2+SEMA4A+, CD34+MFAP5+, and CXCL10+CCL19+. Among those clusters SPARC+COL3A1+ and CXCL10+CCL19+ were significantly expand in all the inflamed but not normal tissues (44) (66). Gene ontology (GO) and transcription factor analysis of the SPARC+COL3A1+ indicated enriched expression of the ECM and Notch signalling pathways, whereas CXCL10+CCL19+ was enriched in genes regulating lymphocytes chemotaxis, T cell proliferation, NF-κ B and interferon (IFN) signature. The interaction between SPARC+COL3A1+ and endothelial cell *via* NOTCH has been demonstrated *in vitro*, consolidating the role of endothelial/fibroblasts crosstalk in priming synovial tissue to increase lymphocyte infiltration. This SPARC+COL3A1+ population closely corresponds with NOTCH-activated THY1+ SFs during RA (6, 8, 36, 69). SPARC+COL3A1+ fibroblasts might expand before CXCL10+CCL19+, suggesting a later differentiation in favour of lymphocyte interaction after endothelial activation. Additionally, HLA-DR^hi^ synovial fibroblasts might be related to the CXCL10+CCL19+ due to their strong IFN response. Recent evidence supports the concept that SFs-induced CXCL10 expression is achieved following stimulation by TNF-α and IFN, which subsequently activated T cell *via* CXCR3 (70). Overlapping activation states of fibroblasts is found across tissues and diseases but may not be exclusive to inflammation. Indeed, a recent study identified the crosstalk between epithelial cells and fibroblasts through SPARC promoting a chronic wound healing phenotype in idiopathic pulmonary fibrosis (71). This highlights that the state of fibroblast activation is potentially responsible for driving disease by communicating with different cell types through similar signals. In summary, integrative clustering studies on stromal cells has revealed new approaches to understand common cellular activation mechanisms leading to potential cross-disease therapies (**Figure 2**).

## 4.0 Fibroblasts and ageing

### 4.1 Cellular senescence of fibroblasts

Cellular senescence, irreversible cell growth arrest, was initially documented in 1961 by Hayflick and Moorhead with their experiments on primary human fibroblasts, which demonstrated a finite proliferation lifespan in vitro and a gradual exhaustion of their replicative potential (72). The subsequent identification of senescent cells in evolutionarily conserved embryological organs through major animal lineages revealed their phenotypic ancestry and gave rise to several theories regarding their contribution to ageing (73–78). Since then, senescence has been shown to be a complex phenomenon with beneficial functions in embryonic development (73, 75, 79), wound healing (80–82), tissue repair (83) and cancer prevention (84, 85), but also critical deleterious roles in organismal ageing (86, 87), age-related diseases (87), abnormal immune responses, inflammation, and even tumorigenesis primarily detected later in life (88, 89). It has been proposed that ageing is caused by the depletion of stem and progenitor cells alongside the effects of senescent cells’ inflammatory phenotype (84, 90). Comprehensive research has been conducted to reveal the molecular triggers, mechanisms, and functionality underpinning senescent cells in tissues and how these contribute towards ageing and age-related diseases.

Cellular senescence refers to the genetically programmed and stable proliferation arrest state that cells undergo in response to detrimental stimuli (89, 91, 92). These stimuli, arising from intrinsic and extrinsic stresses, limit the propagation of damaged and stressed cells, causing them to enter a permanent cell cycle pause state (93). In quiescent skin fibroblasts, permanent senescence is induced by cell-autonomous (cell cycle exit), non-cell autonomous (ability to modulate surrounding tissues) as well as exogenous (e.g. tobacco smoking, UV irradiation) stressors, leading to various responses (94–96). Telomere attrition, or so-called telomere shortening, one of the first molecular senescence triggers reported, is caused by the progressive shortening at the chromosomal ends with repeated cell divisions (97, 98). This is characteristic of fibroblast senescence and is referred to as replicative senescence (RS), induced by replicative exhaustion (72, 93). Additionally, senescence in dermal fibroblasts is marked by mitochondrial dysfunction, also known as mitophagy (99–102); DNA damage and subsequent activation of the DNA damage response (DDR) signaling pathway (103, 104); loss of proteostasis caused by the dysregulated homeostasis of the cellular proteome driven by aberrant protein synthesis, folding and degradation (105–107); excessive oxidative DNA damage and oxidative stress (102); chromosomal and epigenetic aberrations, including post-translational modifications like cross-linking and oxidation (107); metabolic reprogramming and impairment in the DNA repair mechanisms (107, 108).

These triggers provoke the upregulation or downregulation of corresponding proteins, driving fibroblast senescence, which enhance tissue ageing. Mitochondrial dysfunction, for example, associated with the formation of reactive oxygen species (ROS) in ageing fibroblasts, amplifies the expression of activation-protein 1 (AP-1) and NF-κB dependent signaling pathways leading to excessive DNA damage in the skin and elevated accumulation of superoxide ions, which have been indicated as inducers of fibroblast senescence and accelerated skin ageing (109–111).

It is also well-established that elevated cyclin-dependent kinase (CDK) levels are another common feature of cellular senescence in dermal fibroblasts (107). Among those, p16^Ink4a/Arf^ (CDKN2A) and p21 (CDKN1A), two of best well-known senescence markers, alongside the tumor suppressor protein p53 and the senescence-associated β-galactosidase (SA-β-gal), associated with lysosomal activity, present a significantly increased expression during fibroblast senescence, playing a causal role in skin ageing and age-related diseases (85, 87, 112–114). Importantly, clearance of p16^Ink4a/Arf^ positive senescent cells using senolytic treatment in both BuRB1 progeroid ATTAC transgenic mouse models reverses the effects of senescence in tissues and prevents the accumulation of age-related diseases (87, 112). Besides that, fibroblast senescent cells also exhibit enhanced phosphorylated γ-H2AX, a DNA Damage Response (DDR) marker, the inactivation of which causes senescent cells to resume DNA replication (113, 115, 116).

### 4.2 Tissue ageing: The role of fibroblasts

The ability of the human body to resolve inflammation decreases with advancing age, causing an imbalance between pro-inflammatory and anti-inflammatory cellular profiles (117). This gives rise to “inflammaging” - the chronic low-grade pro-inflammatory state that accelerates with older age and is predominantly observed in age-related diseases, such as osteoarthritis, cardiac disease and even several tumorigenesis types as well as other non-age related disease like diabetes (117, 118).

Tissue ageing is characterized by the systemic increase in multiple pro-inflammatory cytokines caused by the accumulation of senescent cells (117). Although these are stable in cellular division and growth, they remain viable and metabolically active, implementing a complex response known as the senescence-associated secretory phenotype (SASP) (119, 120), characterized by the secretion of a range of pro-inflammatory molecules including chemokines (macrophage inflammatory proteins (MIPs), monocyte chemoattractant proteins (MCPs)), interleukins (IL-1, IL-6, IL-8, IL-18), receptor ligands, matrix metalloproteases (MMPs), ECM components, growth factors (transforming growth factor β (TGF-β)), proteases, and cytokines, that can induce inflammation, alter the microenvironment and modulate neighboring cells (121–123).

Previous studies have documented that SASP is the primary mechanism by which senescent cells detrimentally impact on the skin and other tissues (124). SASP is required for the clearance of senescent cells, which they accomplish by recruiting and secreting immune factors that initiate phagocytosis (91). Therefore, it plays a critical role in directing cells of the innate and adaptive immune system towards fibroblast senescent cells, promoting their clearance, and terminating inflammation (124). However, aged tissues accumulate excessive senescent cell numbers contributing to age-related tissue deterioration (125). This feature, along with impaired immune activities, stem and progenitor cells exhaustion observed with advanced age, results in the accumulation of persistent senescent cells that aggravate damage and contribute to tissue ageing (91). For instance, inefficient clearance leads to loss of homeostasis and imbalance between senescence and clearance, resulting in deficiencies and age-related diseases (90, 126, 127). Previous research has shown that Nrf2, a redox regulator used to induce senescence *in vitro* and *in vivo*, creates an aberrant ECM when overexpressed in fibroblasts (128). This aberrant ECM, rather than promoting wound healing, acts in a pro-tumorigenic manner, suggesting that the beneficial effects of senescent cells are context-dependent (94). Therefore, if not cleared from the tissue, senescent cells can dominate and cause detrimental effects (94).

Senescent fibroblasts with an abnormal SASP can also disrupt the interactions between the dermis and the epidermis (129). Fibroblasts derived from the dermis release an insulin-like growth factor (IGF-1) which is essential for the mesenchymal stem cell niches (MSC) and regulates the balance of epidermal cell proliferation and differentiation (129). During fibroblast senescence, IGF-1 signaling is downregulated because of enhanced superoxide ion production accumulating from the dysfunctional mitochondria of the aged human and murine dermal fibroblasts (130). Later evidence indicates that deprived IGF-1 and inhibition of the relaying IGF-1 signal causes suppression of collagen synthesis in the dermis and subsequent epidermal atrophy due to enhanced DNA damage induction, γH2AX and p16^Ink4a/Arf^ production in the epidermal cells, consolidating the data underlying the vitality of IGF-1 in the human skin (129, 131, 132).

It is also vital to note that, the pathophysiology and inflammaging of the skin is a multifactorial rather than a single event, created by the complex network of cellular cross-link communication among fibroblasts, keratinocytes and melanocytes, as well as their interactions with the external environmental stressors (117).

Interestingly, besides the production of pro-inflammatory cytokines and interleukins derived by SASP, recent evidence highlights the role of inflammation in fibroblast senescent cells’ generation from an alternative, intriguing ankle. For instance, data from metabolically downregulated C3-/- mice, injected with monosodium urate signals (MSU), present fibroblast senescence, which is elevated in aged-cultured cells, marked by enhanced expression of senescence-associated β-galactosidase, as well as p15, p16 and p21 senescent markers (53). Therefore, when the complement 3 component of the innate immune system is abolished, fibroblasts cannot be primed and become senescent (53). Similar effects are also observed when mTOR and HIFα are pharmacologically inhibited using rapamycin and the translational inhibitor KC7F2, respectively (53). The authors, therefore, propose that senescence induction *via* pharmaceutical inhibition of mTOR or HIFα, or C3 depletion, create an immune-regulatory phenotype, prohibiting uncontrolled complement activation, ameliorating the detrimental inflammatory effects recurring at specific previously affected sites (53). However, it would also be beneficial to determine whether this C3 abolishment creates any adverse effects in the long term or measure the exact levels of senescence that create this anti-inflammatory effect.

Senescence is also induced *via* activation of the melanocortin type 1 receptor (MC1), which alternates the genetic expression, increasing MMP expression, deteriorating collagen production and altering the remodeling phase of wound healing. Nevertheless, recent evidence shows that selective agonism of MC1 through activation of G-protein coupled receptor (GPCR) and dependence on the ERK1/2 downstream phosphorylation ameliorates inflammation in the joints and protects the cartilage *via* inducing senescence in the synovial tissue (133). Therefore, selective MRC1 expression inducing senescence is proposed as a promising therapeutic target against inflammatory arthritis (133).

This evidence thus indicates the vitality of harmonized senescence fibroblasts, strengthening the hypothesis that the absolute depletion of senescent cells can also be detrimental to the tissues.

### 4.3 Fibroblast heterogeneity during ageing

Biological ageing can be considered as a disease on its own (134). Ageing leads to the accumulation of degenerative biological processes involving a progressive decline in organismal homeostasis (135). By driving modifications in the tissue, including increased inflammation, loss of tissue regeneration, fibrosis, and metabolic imbalance, ageing is a major comorbid factor in many diseases. While cellular senescence participates in many processes, other ageing factors that drive age-related disease are not yet fully understood. To address this issue, several scRNA-seq analysis have tried to elucidate the transcriptional heterogeneity of stromal cells in ageing. A study on mouse dermis revealed that old fibroblasts exhibit transcriptional noise and partially lose their identity while adopting adipocyte characteristics (136). A GO analysis comparing young and aged dermal fibroblasts indicated an upregulation of genes related with the immune response, a characteristic of the ageing cells (24). These mechanisms are not skin exclusive, as old murine cardiac fibroblasts also upregulate pathways associated with inflammation or ECM regulation. Interestingly, old cardiac fibroblasts seem to upregulate genes related to osteogenesis, indicating their potential involvement in epicardial layer calcification observed in elderly individuals (137). These data suggest that ageing fibroblasts may lose their specialized identity leading to abnormal functions. However, fibroblasts may not converge toward a common ageing phenotype, suggesting that tissue specificity remains. Future single-cell transcriptomic studies on other tissues and diseases will help to elucidate a potentially shared aging phenotype.

## 5.0 Therapeutic perspectives of targeting fibroblasts in inflammation and ageing

Many efforts have been made to control the pathological phenotype of fibroblasts in different tissues in order to develop new approaches for the treatment of several diseases. In this section, we discuss the strategies that have been employed to develop a fibroblast targeted therapy.

Targeting cytokines, that are involved in the impaired functions of fibroblast, is considered of value for managing arthritis and other fibroblast-related conditions. Current therapeutic strategies that block fibroblast-activating signals such as anti- TNF therapy and anti- IL-6 receptor blocking antibodies, have shown clinical relevance for the treatment of chronic inflammatory conditions (138). Moreover, a range of strategies (such as monoclonal neutralising antibodies and small-molecule inhibitors) to treat cancer and fibrosis have been developed against TGF-β1; highly expressed and produced by fibroblasts (139, 140). However, targeting a single stimulus is not considered an efficient approach in blocking fibroblast activation with the presence of other stimulating factors. Moreover, this approach can prevent the activation of other cell types and induce side effects under certain conditions (3, 139). For this reason, intracellular proteins and signalling pathways, such as MAP and JAK (Janus kinase), have also attracted attention as targets to control fibroblast activation in RA regardless of stimulator type (141). The inhibition of transcriptional activators YAP/TAZ in fibroblasts is also considered as a promising strategy to explore for RA therapy. Indeed, the suppression of YAP/TAZ in SFs led to a reduction in their resistance to apoptosis, their inflammatory phenotype, proliferation, and ability to invade (142, 143). Further research highlights the use of YAP/TAZ inhibitors as a treatment for Crohn’s disease and for controlling intestinal fibrosis (144). In addition, recent findings have demonstrated the advantages of suppressing YAP/TAZ in fibroblasts by activating the dopamine receptor D1 (DRD1) in mouse models of lung and liver fibrosis (145).

These inhibitory approaches are now making progress with several preclinical studies aiming to design small inhibitor molecules with a high degree of selectivity. However, the risk of nonspecific and off-target effects requires deep consideration when developing these molecules. Targeting cytokine signal transduction may not be sufficient, especially in chronic conditions where multiple factors can promote the abnormal behaviour of fibroblasts (146). In this context, epigenetic dysregulation such as histone modification and miRNA expression are also implicated in the pathogenic phenotype of RA FLS (147, 148). Thus, manipulating the epigenetic mechanisms of fibroblasts could be beneficial to develop new drugs for RA and other pathologies. In fact, induction of protective miRNAs expression and inhibition of HDACs (histone deacetylase) as well as BET proteins have shown promising results in RA SFs and animal models of arthritis (149, 150). Beside epigenetic changes, miRNAs can target specific pathways involved in disease progression. For instance, miR-17 supress the IL-6 family autocrine loop by targeting JAK1/STAT3 pathway leading to anti-inflammatory and anti-erosion responses (151). Furthermore, inducing MiR-613 reduce the expression of the targeted transcript encoding for DKK1 in RA SFs which altered their proliferation, apoptotic resistance, and aggressiveness (152). On the other hand, targeting dysregulated miRNAs in CAFs might be promising for the development of novel anti- cancer drugs (153, 154). Several studies have also established the beneficial effect of inhibiting DNA methylation and HDACs in the suppression of the pro-fibrotic phenotype by inhibiting the myo-fibroblasts trans differentiation of hepatic stem cells (HSCs) (155), or by directly targeted HSC derived myofibroblasts and their apoptotic resistance (156, 157).

In addition to the epigenetic machinery, strategies that target the imbalanced metabolic pathways in both RA SFs and CAFs have been employed to reduce RA severity and inhibit tumour growth respectively (158, 159)). Interestingly, a recent study proposes a new strategy to control fibrosis and hypertrophic scars formation using an anti-glycolytic agent that regulates fibroblasts activity (160). Glycolysis inhibition has also been shown to reduce cardiac fibroblasts activation *in-vitro* and to control cardiac fibrosis in mice with myocardial infarction (69). Targeted inhibition of glycolysis in fibroblasts is therefore considered as a promising approach for the treatment of several fibroblasts -related diseases.

Cellular senescence is another feature of RA fibroblasts that contribute to the chronicity of arthritis by triggering a pro-inflammatory phenotype (161). However, a recent study introduced fibroblast senescence induction as a new way to control joint inflammation through the selective activation of G-protein coupled receptor, MC1R, that inhibits FLS proliferation and promotes the acquisition of a pro-reparative phenotype with anti-arthritic effects in K/BxN arthritis model (133). Furthermore, senescence inhibition has been shown to have anti-fibrotic effects on IPF fibroblasts. Additionally, some anti-fibrosis and anti-cancer drugs are known to alleviate the senescence of lung fibroblasts (162).

The development of single-cell RNA-sequencing technologies highlighted the presence of distinct subsets of fibroblasts associated with different pathological conditions. Therefore, specific elimination of aberrant fibroblasts based on a specific cell marker may inform new opportunities to investigate novel drug targets in multiple diseases such as fibrosis, cancer, and chronic inflammatory diseases (3) (163). As described earlier, CXCL10+CCL19+ immune-interacting and SPARC+COL3A1+ vascular-interacting fibroblasts were identified as two fibroblast subtypes shared between inflamed tissues of four inflammatory diseases involving lung, intestine, salivary gland, and synovium. These inflammatory clusters are therefore suggested as novel therapeutic targets which may provide new approaches to develop common therapies for multiple chronic inflammatory diseases (66).

Deletion of systemic and local FAPα+ fibroblasts lead to protection associated with reduced leukocyte infiltration, inflammatory mediators, and joint damage in mouse models of arthritis (60). FAP expression on myofibroblasts of mice with IPF permits to selectively target fibroblasts which are promoting tissue fibrosis. Indeed, treatment with FAP-targeted PI3K inhibitor decreased collagen production and deposition and increased mouse survival (164). In this same context, COL1+ fibroblasts have been recently identified as a good tool to test the effect of inhibiting genetically STAT 3 in mouse model of colitis-associated colorectal cancer, where it led to a decreased proliferation of tumor cells (165).

Similarly, targeting NOTCH3 receptor expressed on fibroblasts genetically or therapeutically promotes anti-arthritic effects in mice by significantly reducing joint swelling and bone erosion (61).

Cadherin 11 also represents a potential candidate to control the altered behaviour of fibroblasts in several pathologies such as arthritis and fibrosis. Different strategies such as antibody-mediated blockade and genetic deletion of this surface marker have been tested and shown their effectiveness (166, 167).

All together, these findings suggest that targeting fibroblast specific molecules could be a therapeutic avenue for RA and other chronic diseases that involve fibroblasts. However, specific depletion of pathogenic subset of fibroblasts based on their surface markers without side effects on tissue homeostasis and other organs is challenging (141, 168).

## Conclusions and perspectives

**Figure 3:**
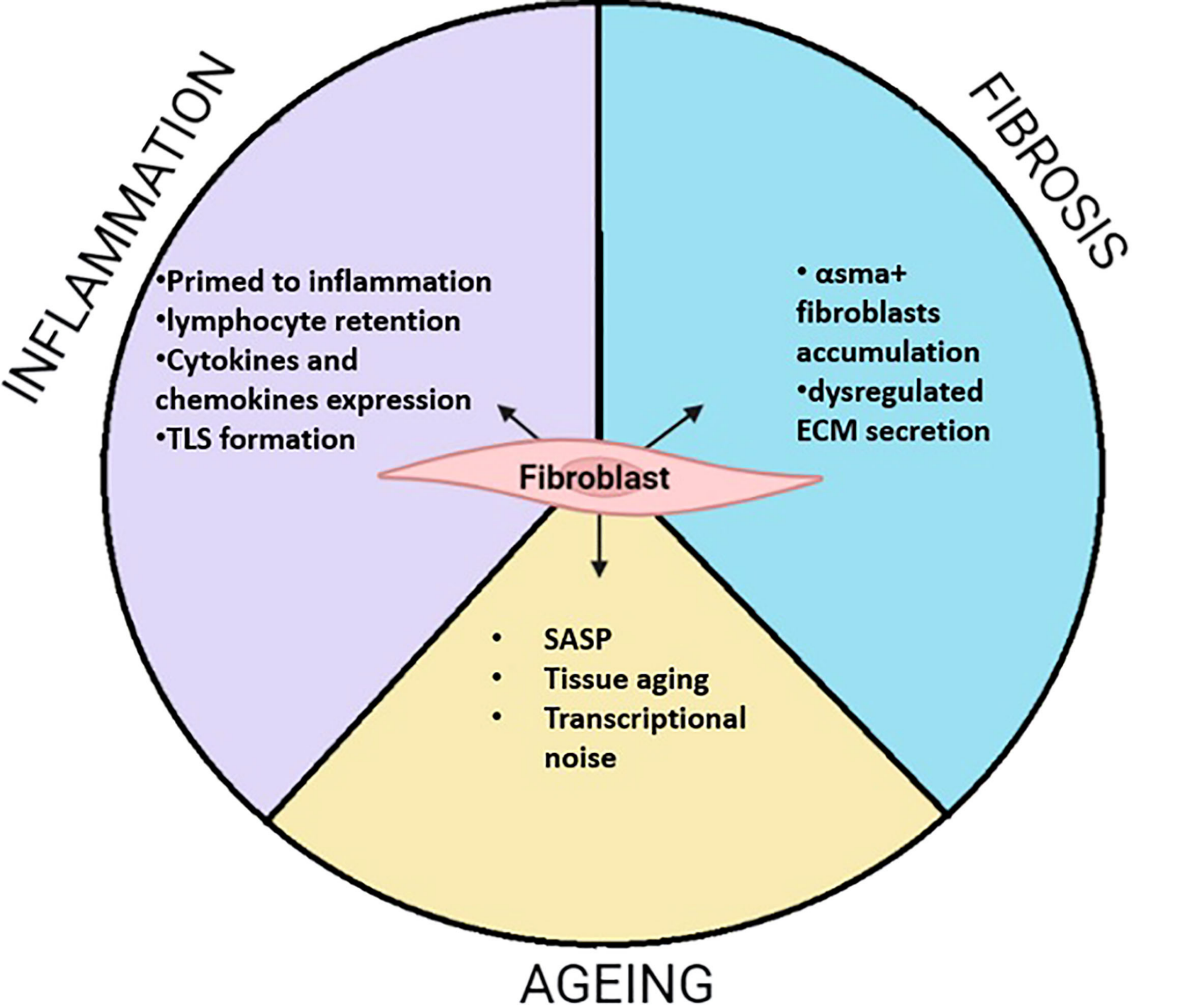
Summarized function of fibroblasts during inflammation, fibrosis and Aging. During inflammation fibroblasts adopt activate phenotypes. This often leads to the priming of fibroblasts which remains potent to inflammatory stimuli. This is characterized by the secretion of pro-inflammatory cytokines and chemokines. Those properties can lead to local accumulation of lymphocytes where fibroblast support their survival by tertiary lymphoid structure (TLS). In fibrosis, my fibroblasts are accumulating and secreting abnormal ECM modules leading to matrix stiffness enabling proper network communication. During aging, fibroblasts highly participate to low grade inflammation call inflammaging through the release of SASP containing pro inflammatory cytokines. Evidence shows that fibroblasts transcriptome changes toward a less define transcriptomic identity called transcriptional noise. While other old fibroblasts, adopt other cell type characteristics such as osteoclasts in cardiac fibroblast or adipocyte in the skin.

From their embryonic origins in primary mesenchymal tissue to their specialized sub population observed in specific niches, fibroblasts have colonized and shaped the entire organism by connecting organs and cells together. Their contribution to ECM secretion is essential to maintain tissue function and create precise networks for cell communication. However, fibroblasts are not just structural cells. Single cell RNA sequencing reveals the transcriptomic heterogeneity of fibroblasts underpinning essential regulation in health, aging and diseases (**Figure 3**). The characterization of fibroblasts subsets and their positional identities in many organs highlight their role in supporting very specific functions within the tissue. However, those processes are perturbed during diseases such as rheumatoid arthritis, pulmonary fibrosis, or ulcerative colitis where specific fibroblast subsets can lead to disease progression. The identification of overlapping sub populations across inflammatory diseases reveals shared mechanism influencing fibroblasts’ behaviours and leading to aberrant inflammatory responses. Communication between endothelial and fibroblasts presents a critical role in the establishment of pathogenic fibroblasts subset which then participate to the recruitment and retention of immune cells, impairing resolution of inflammation. The discovery of common activation mechanisms driving phenotypic changes in fibroblasts across diseases and organs might suggest a plasticity of this cell type. Despite the proven existence of a universal “reservoir” fibroblast population (12), its biological relevance might be difficult to study due to plasticity. However, the potential or limitation of this plasticity needs to be further described particularly when developing induced pluripotent stem cells from fibroblasts (169, 170). Interestingly, recent studies on ageing fibroblasts report that they adopt other cell type traits such as adipocytes in the dermis, or osteoblasts in cardiac tissue. In contrast, fibroblast-driven inflammation is common during disease or ageing. While pathogenic sub populations emerge after repeated inflammatory stimuli, the ageing processes also induce changing in the aged fibroblasts leading towards inflammation. However, those changes do not increase heterogeneity in distinct populations but rather in stochastic transcriptional noise. One can therefore suggest that tissue ageing acts as a potent environmental cue driving fibroblasts to an increasingly common inflammatory phenotype and loss of their tissue distinct expression patterns. To address this, the transcriptomic profiles and functional characteristics of fibroblasts need to be further investigated across a range of tissues in both health and disease. Finally, the identification of transcription factors regulating the fate of fibroblasts toward a destructive or pro fibrotic phenotype remains unclear. The recent evidence of ETS1 as a transcription factor inducing ECM degradation programme in SFs leads to a better understanding the opposing role of fibroblasts in destructive/inflammatory diseases compared to pro fibrotic pathologies (171). Interestingly, the transcription factor PU.1 has been identified as a regulator of pro fibrotic phenotype in fibroblasts (172). Moreover, ETS1 and PU.1 are mutually exclusive, and their expression does not overlap in fibroblasts in both IPF and arthritic synovium (171). This highlights an important fate decision mechanism in fibroblasts by driving a pro destructive or pro fibrotic phenotype. Nonetheless, the mechanisms that control those fate decision pathway remains unexplored.

## Author contributions

VG and MK contributed equally. All authors contributed to the article and approved the submitted version.
